# Factors affecting weight and body composition in childhood cancer survivors—cross-sectional study

**DOI:** 10.3332/ecancer.2020.999

**Published:** 2020-01-13

**Authors:** Małgorzata Sawicka-Żukowska, Włodzimierz Łuczyński, Jakub Dobroch, Maryna Krawczuk-Rybak

**Affiliations:** Department of Pediatric Oncology and Hematology, Medical University of Białystok, 17 Waszyngtona Street, 15-274 Bialystok, Poland; a http://orcid.org/0000-0001-5437-7119

**Keywords:** paediatric oncology, child cancer survivor, obesity, fat tissue

## Abstract

Due to improved efficacy of antitumour treatment in the general population, there are increasingly more childhood cancer survivors. However, some of these survivors are at risk of distant complications including cardiovascular disease. We aimed to examine the risk of overweight/obesity and abnormal body composition in a large group of patients from our paediatric oncology centre. We used anthropometric methods and electrical bioimpedance to assess these features, and then determined their association with disease and treatment. We found patients treated for leukaemia/lymphoma (especially boys) had significantly higher rates of overweight/obesity compared to the other patient groups. On the contrary, overweight/obesity was more common in girls among patients treated for solid tumours. Patients treated for leukaemia/lymphoma were characterised by a higher body fat content compared to those treated for solid tumours and controls. During treatment for cancer, patients had a higher percentage of muscle mass deficiency compared to those in the control group. Our regression analysis showed time from completion of treatment, gender and type of therapy (radiotherapy, megachemotherapy) were associated with body weight and body composition including fat and muscle content. We recommend paediatricians and general practitioners should actively try to detect and prevent cardiovascular disease among childhood cancer survivors.

## Background

The efficacy of comprehensive antitumour treatment has improved over the years, leading to an increase in the number of childhood cancer survivors. Nonetheless, cancer survivors should be monitored for relapse of underlying disease, secondary tumours, and late complications of therapy, including endocrine complications such as obesity and diabetes [1, 2]. Indeed, the risk of obesity was shown to be higher among cancer survivors than their healthy siblings [3]. Persons with central nervous system (CNS) tumours, those treated for acute lymphoblastic leukaemia (ALL), and patients after transplantation of hematopoietic cells are especially at risk for this type of complication [4]. Cardiovascular disease, obesity and diabetes are one of the main responsible for increased mortality and morbidity among childhood cancer survivors (reviewed in [5]). The task of detecting these complications falls to family physicians, paediatricians and paediatric oncologists and the patients themselves.

To identify these complications diverse measures are used. The most universal marker for the diagnosis of overweight and obesity is the body mass index and standardised deviation score (BMI-SDS); its calculations should be based on updated country norms [6]. For assessment of fat and muscle mass content, bioelectrical impedance analysis (BIA) is used. The results obtained from BIA correlate with data from the reference dual-energy X-ray absorptiometry (DXA) method [7, 8]. BMI is very easy to assess, but it is an anthropometric parameter and therefore should be considered as a surrogate measure for fatness. Although BMI correlates with percentage body fat (PBF) assessed by BIA or DXA, the correlation between both parameters is not sufficiently accurate to truthfully reflect the amount of fat in the body in a particular subject. Therefore, if fatness is the true risk factor for longevity and health, then BMI is only an approximation and is, therefore, inadequate [9]. On the other hand, BIA results can be affected by hydration level, skin temperature, exercise, the use of diuretics, female menstruation, and the need to void the bowel and bladder before the assessment.

The studies carried out so far concerning cardiovascular disease risk factors in childhood cancer survivors have typically involved small groups of patients, or patients with one diagnosis, and typically use only BMI or BIA/DXA for assessment of obesity and excessive body fat. With this in mind, in our cross-sectional study, we aimed to identify clinical factors that affect body weight and body fat and muscle content in people who are currently treated or have had cancer in childhood. We examined body weight and body fat and muscle content in a large group of patients from our paediatric oncology centre using anthropometric methods and electrical bioimpedance. We then examined the relationship(s) of these factors with features associated with the disease and/or its treatment. The data obtained may be used in the future to assess the health condition of childhood cancer survivors.

## Patients and methods

### Patients

This study was carried out at the Department of Paediatric Oncology and Haematology at the Medical University of Bialystok, Poland from 2014 to 2017 (approved by the Bioethics Committee, consent number: 153-798883L). It was designed as a cross-sectional study evaluating body mass and bioimpedance during and after cancer treatment in childhood. The tests were performed during regular follow-up visits in all patients who met the inclusion criteria. The study inclusion criteria were: diagnosis and treatment of cancer according to the Polish Group for the treatment of leukaemia/lymphomas and solid tumours criteria; Polish nationality; aged 2–22 years; good general condition at the time of the study (according to the physical examination); consent of the parents/guardians and/or the patient themselves for inclusion in the study; and in post-treatment patients, remission of the underlying disease. Study exclusion criteria were: relapse or progression of the disease, chromosomal disorders, mental disorders (including eating disorders such as anorexia, bulimia), hormonal and autoimmune disorders including thyroid and adrenal gland disorders, celiac disease, and diabetes. The control group (aged 4–23 years, matched in terms of age and sex with the group of patients after cancer treatment) was composed of siblings/families of patients with cancer, volunteers, and children admitted to the clinic for reasons other than cancer. The control group included patients in whom cancer, chromosomal, autoimmune, hormonal, and psychiatric disorders were ruled out. Parents/guardians and/or subject themselves provided consent for inclusion in the study.

The study group was divided into the following four subgroups, according to the type of cancer and whether they were undergoing active treatment or not: 1) patients currently undergoing intensive/maintenance treatment for leukaemia or Hodgkin’s/non-Hodgkin’s lymphoma, 2) patients after completion of treatment for leukaemia/lymphoma (>1 year after therapy termination), 3) patients currently undergoing treatment for solid tumours and 4) patients after completion of treatment for solid tumours (>1 year after therapy termination). Solid tumours group included patients with rhabdomyosarcoma, Langerhans cell histiocytosis, neuroblastoma, osteosarcoma, germ cell tumours, Wilms tumour and Ewing Sarcoma. Patients treated with steroids were considered those who were receiving a dose of 60 mg/m^2^ daily of prednisone or equivalent for a minimum of 30 days. Radiation therapy involved a minimum dose of 12 Gy and may have been applied to the following regions: CNS, neck, mediastinum, abdominal cavity and/or total body.

### Anthropometric methods

Anthropometric parameters including age, sex, height, weight, and BMI-SDS were evaluated in all subjects. The disease-associated parameters evaluated in the group of cancer patients/survivors were: type of cancer, treatment regimen used, elapsed time since therapy termination, use of radiotherapy, history of steroid treatment and history of allogeneic hematopoietic stem cell transplantation (HSCT).

The examination included measurements of height (cm, Harpenter’s stage-meter) and weight (kg) conducted by trained members of the study team. BMI (kg/m^2^) and BMI-SDS values were calculated and coded as follows: underweight = BMI-SDS < −1; normal weight = −1 ≤ BMI-SDS < +1; overweight = +1 ≤ BMI-SDS < +2; obesity = +2 ≥ BMI-SDS [1].

### Bioelectrical impedance analysis

BIA was used to assess the body composition (device: InBody 370 Biospace, USA). All tests were performed by two trained members of our team (Małgorzata Sawicka-Żukowska and Jakub Dobroch). Body fat content in grams (FAT), PBF, and skeletal muscle mass (SMM) were chosen among the parameters obtained from the device. All these parameters were referenced to age and gender norms entered into the device software; thus, each result was given as below, normal, or above the norms.

### Data presentation and statistical analysis

Data are presented as means and standard deviation (SD) or medians and interquartile range (IQR), and rates of incidence of a given characteristic in the evaluated group of children. Due to the non-normal distribution of most of the variables, non-parametric tests were performed. To find the differences between the groups of patients and controls ANOVA was performed and *post-hoc* pair-wise comparisons. Correlations were performed using Spearman’s test. Multivariate linear regression was used to evaluate the impact of clinical features on BMI-SDS, PBF, and SMM. A *p* < 0.05 was considered statistically significant. Statistical analysis was made in Statistica 13 (Dell, USA).

## Results

The size of the individual patient groups and the distribution of gender and age are presented in Table 1. Children currently undergoing treatment for leukaemia/lymphoma or solid tumours were significantly younger than those in the other groups (Table 1). The median time from the end of anticancer treatment was significantly shorter in patients treated for leukaemia/lymphoma than in those treated for solid tumours (Table 1).

### Changes in BMI

The BMI-SDS and the incidence of normal/abnormal bodyweight composition in each group are summarised in Table 2. Patients who had completed treatment for leukaemia/lymphoma were characterised by significantly higher BMI-SDS than the other patient groups. Likewise, patients with leukaemia/lymphoma (both during and after treatment) had statistically significantly higher overweight/obesity rates compared to the control group and those with solid tumours (Table 2).

We found different BMI-SDS and overweight/obesity rates among patients with cancer according to gender. After treatment for leukaemia/lymphoma, overweight/obesity were more common among boys than girls (50.0% versus 35.0%, respectively; *p* < 0.01). However, the opposite relationship was observed in patients following treatment for solid tumours: overweight/obesity was more common in girls than boys (21.2% versus 7.5%, respectively; *p* < 0.01).

The BMI-SDS was then analysed in groups of patients after antineoplastic treatment according to the time from the end of therapy. Within 5 years after the end of treatment for both leukaemia/lymphoma and solid tumours, patients had a higher BMI-SDS and higher overweight/obesity rates; however, these differences were not statistically significant. Such relation was not observed after 5 years on completion of therapy.

Further analyses were made by studying the effects of therapy on the current BMI-SDS and overweight/obesity rates. After treatment including any type of radiotherapy, patients with leukaemia/lymphoma were characterised by significantly higher BMI-SDS and more cases of overweight/obesity than those treated without any irradiation (medians 1.34 versus 0.46; *p* = 0.02; cases: 19.3/33.8% versus 13.2/25.6%; *p* = 0.04, respectively). There were no statistically significant differences in BMI-SDS values and overweight/obesity rates in patients treated for leukaemia/lymphoma with or without allogeneic HSCT.

### Bioelectrical impedance analysis

The percentage of patients with normal and abnormal amount/percentage of fat and muscle is shown in Table 3. Patients with leukaemia/lymphoma (both during and after treatment) were characterised by higher body fat content compared to those following treatment for solid tumours and control subjects (Table 3). Patients currently undergoing treatment for leukaemia/lymphoma and solid tumours had higher rates of muscle mass (SMM) deficiency compared to those in the control group (Table 3).

The above variables were subsequently analysed according to gender. During treatment for leukaemia/lymphoma, girls showed greater mean fat mass percentage compared to boys (PBF 71.8% versus 61.5%), as well as compared to girls and boys in the control group (30.7% versus 29.7%). However, after treatment for leukaemia/lymphoma, boys had a higher fat mass compared to girls (the proportion of results above the norm was 55.6% in boys versus 36.3% in girls). After the treatment of solid tumours, girls were more likely to have an excessive fat amount compared to boys (PBF 42.4% versus 20.0%).

Further analysis was made by dividing the patients after antineoplastic treatment into subgroups according to the time elapsed since the end of therapy. After treatment for solid tumours, there was a significantly higher proportion of patients with excessive body fat, i.e. 25% in the ≤5 years group compared to 10.4% in the >5 years group (*p* < 0.01). A similar ratio was found in PBF (41.6% versus 25.0%, *p* < 0.01). Regarding muscle tissue in patients after treatment for leukaemia/lymphoma, its deficiency was observed more frequently in patients with ≤5 years since completion of therapy compared to >5 years (48.5% versus 31.8%, respectively, *p* = 0.01); inverse relationships were noted in patients after treatment for solid tumours (SMM deficiency ≤5 years versus >5 years: 29.1% versus 56.2%, respectively, *p* < 0.01).

The effects of irradiation and megachemotherapy with allogeneic HSCT on body fat and muscle content were also analysed. After treatment for leukaemia/lymphoma, the fat mass was higher in patients treated with any type of radiotherapy compared to those treated without radiotherapy (variables normalised to age and gender): excess of FAT was observed in 40 (64.5%) patients irradiated and 47 (38.8%) patients without irradiation (*p* < 0.01); excess PBF was found in 47 (77.0%) patients and 66 (54.5%) patients with and without irradiation, respectively (*p* < 0.05). The proportions were similar in patients after treatment of solid tumours, but were only statistically significant for PBF: 14/37.8% versus 8/22.2% with and without irradiation, respectively (*p* < 0.05). Regarding muscle mass (SMM), in the group of patients after treatment for solid tumours, the proportion of patients with muscle mass deficiency increased among irradiated subjects [22/37 (59%) versus 12/36 (33%) patients, respectively; *p* < 0.05].

Megachemotherapy with allogeneic HSCT resulted in a greater fat mass in patients after treatment for leukaemia/lymphoma than those without HSCT (FAT excess: 29/51 [56.8%] patients with HSCT versus 58/132 [43.9%] patients without HSCT; PBF excess: 38/51 [74.5%] versus 75/132 [56.8%], respectively; *p* < 0.01). In patients after treatment with megachemotherapy for leukaemia/lymphomas, higher rates of muscle mass deficiency was observed compared to patients treated without this method, but these differences were not statistically significant.

### Factors influencing the variability in BMI and fat and muscle mass

The factors influencing the BMI-SDS and body fat and muscle mass were analysed in patients following anticancer treatment. The age of the patients was considered as confounding factor. Only statistically significant correlations with probable clinical significance were examined.

**Figure 1.**
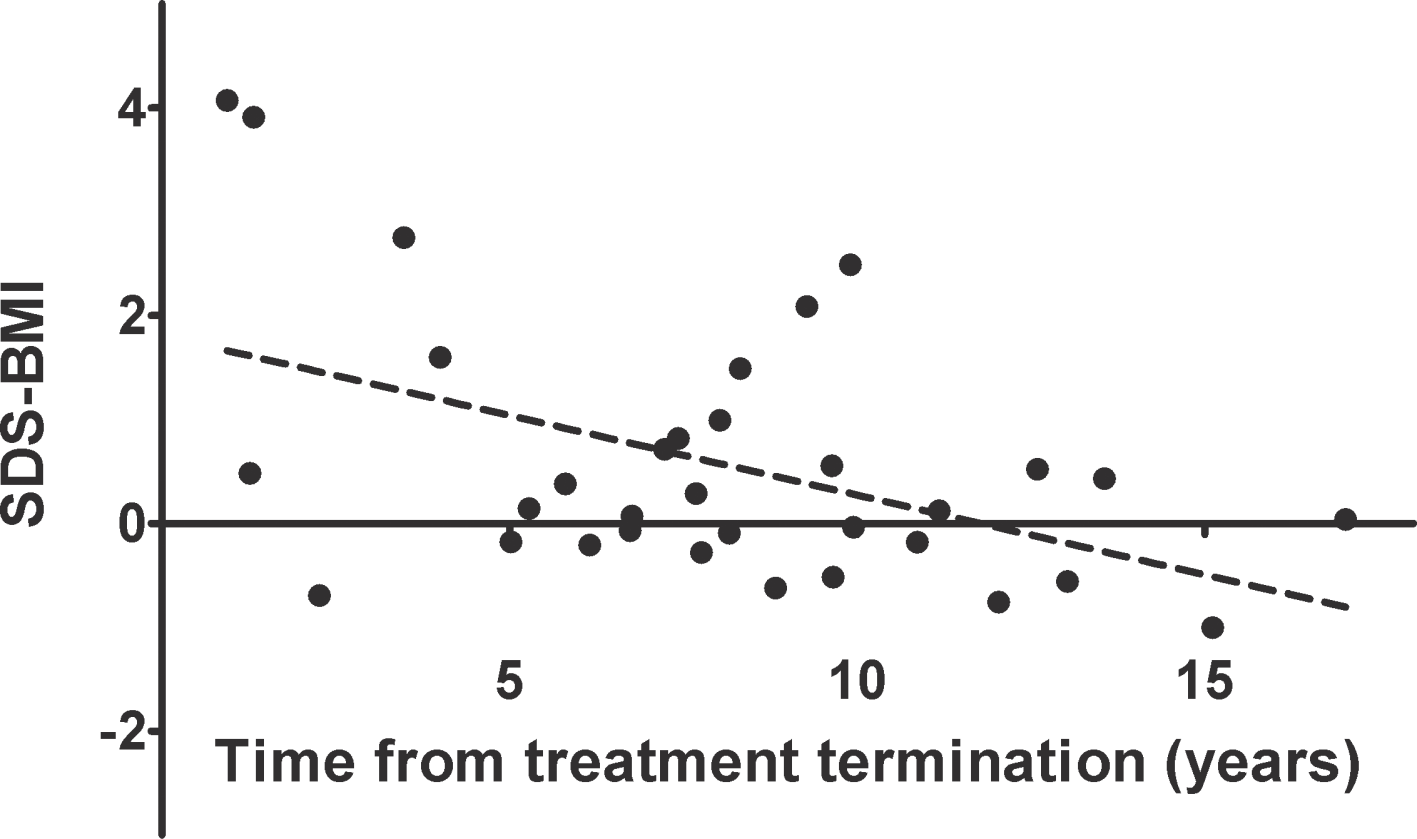
The significant, negative correlation between standardised BMI and time from treatment termination for solid tumours in females (*r* = −0.42, *p* < 0.05).

**Figure 2.**
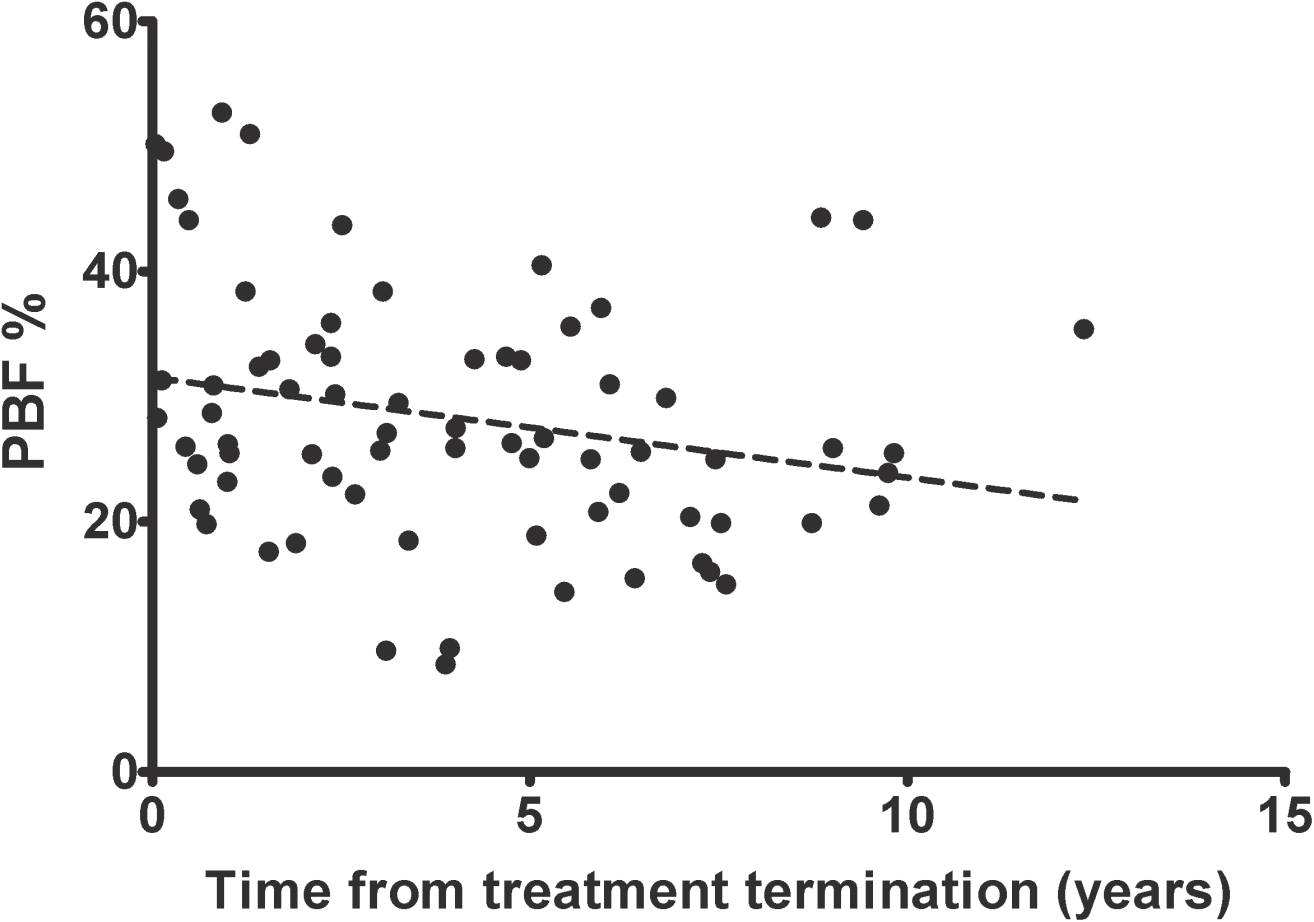
The significant, negative correlation between the percentage of fat mass (% PBF) and time from treatment termination for leukaemias/lymphomas in females (*r* = −0.28, *p* < 0.05).

Following treatment for solid tumours, there was a negative correlation between time since the end of therapy and BMI-SDS (*r* = −0.24; *p* < 0.05). Interestingly, the correlation was stronger after the grouping of patients according to gender (*r* = −0.42 in females and *r* = −0.38 in males, *p* < 0.05; Figure 1). This means the BMI-SDS decreased over time since the completion of antineoplastic treatment in this group of patients. However, we did not find a similar relationship in patients after treatment for leukaemia/lymphoma, even after considering gender. In contrast, the PBF decreased with time after completion of therapy in both groups, but the correlation was only statistically significant after treatment for leukaemia/lymphoma (*r* = −0.28 in females and *r* = −0.22 in males, *p* < 0.05; Figure 2).

There was a positive correlation between SMM and time from treatment completion both after treatment for leukaemia/lymphoma and for solid tumours (*r* = 0.37 and *r* = 0.23, respectively; *p* < 0.05). However, taking into account the patient’s age at the time of the study, the correlation remained positive only in the group after treatment for leukaemia/lymphoma (*r* = 0.21): the correlation became negative in those after treatment for solid tumours (*r* = −0.26; *p* < 0.05). Thus, muscle mass increases over time after treatment for leukaemia/lymphoma and decreases after treatment for solid tumours.

We then developed regression models to explain the variation in body weight, as well as fat and muscle mass. The time from treatment completion and gender explained 46% of the variability in BMI-SDS after solid tumour treatment (*R*^2^ = 0.46, *p* < 0.01). The time from treatment completion, irradiation, gender and age of diagnosis of cancer explained 29% of the variability in PBF after treatment for leukaemia/lymphoma (*R*^2^ = 0.30, *p* < 0.01). A similar model with time from completion of therapy and gender explained 63% of the variability in PBF after treatment for solid tumours (*R*^2^ = 0.64, *p* < 0.00); meanwhile, gender and the use of any type of irradiation explained 49% of the variability in FAT in this group of patients (*R*^2^ = 0.50, *p* < 0.00).

We also performed a regression analysis in a combined group of patients after treatment for both leukaemia/lymphoma and solid tumours (256 patients in total). Only the time elapsed from the end of treatment explained the variability in the BMI-SDS in this group (*R*^2^ = 0.16, *p* < 0.01). The time from the treatment completion and gender explained the variability in PBF (*R*^2^ = 0.33, *p* < 0.00). Finally, the time from the end of treatment, gender, the use of HSCT, the irradiation and the age of cancer diagnosis explained muscle content after treatment for leukaemia/lymphoma or solid tumours (*R*^2^ = 0.84, *p* < 0.00).

## Discussion

The enormous success that has occurred in the treatment of childhood cancer can be diminished by the late effects of therapy. One such complication may be a cardiovascular disease caused by, among others, excessive weight gain and body fat increase. In our study, we identi fied gender, type of cancer, type of treatment, and time from treatment completion as factors affecting the body weight and composition of a large group of patients both during and after the end of childhood cancer treatment.

In our study, we found higher rates of overweight/obesity and excessive body fat after complex treatment for leukaemia and lymphoma among boys; however, after treatment for solid tumours, higher rates were observed in girls. Indeed, the effect of gender on the occurrence of cardiovascular risk factors after the treatment of cancer has been diverse. For example, in a large group of Danish survivors, obesity was more common in the female population, and predisposing factors were younger age, high BMI at diagnosis, and cranial radiotherapy [10]. In another observation, many years after treatment for ALL, young men had a higher proportion of obesity and fat content compared to age-matched controls [11]. Meanwhile, Blijdorp *et al* [12] made interesting observations when evaluating patients after cancer treatment: BMI was higher in women, irrespective of time since treatment, as opposed to men where it increased with time after the end of treatment. In addition, the percentage of fat was only higher than controls in men. Furthermore, as in our study, in a group of non-Hispanic white survivors, 12 years after diagnosis of leukaemia/lymphoma, men and not women had a higher fat content including trunk fat compared to the control group [13]. Most reports indicate that cranial radiation is the cause of the body composition and weight disorders in patients after treatment for ALL, but this type of treatment is becoming less commonly used in standard ALL therapy. However, another study showed women after ALL treatment without cranial radiation did not differ in BMI and body fat from control subjects, and in men, there was no difference between groups after treatment with and without radiation [14]. Nonetheless, for greater reliability of such results, it is necessary to stratify large groups of patients according to multiple clinical features including the type of the disease and therapy.

Of the many types of cancers that occur in children, leukaemia and lymphoma seem to be the biggest risk factors for overweight. This is likely due to the use of glucocorticoids, as well as to irradiation of both the CNS and in HSCT. In a prior analysis of persons after ALL treatment, the main factor affecting the current body weight was body weight at diagnosis and its increase during therapy [15]. In a similar group of patients, only initial weight was a predictor of overweight/obesity after treatment, not age at diagnosis, gender, or the dose of steroids [16]. It should be kept in mind that the effect of the initial BMI on the distant values of this indicator is so great that, in such an analysis, other factors may not have statistical significance [10]. Therefore, in our regression analysis, we did not include the initial BMI-SDS as a factor influencing its current values.

Traditionally, it is assumed that patients with solid tumours, even after treatment, have fewer problems with overweight/obesity than those with ALL, mainly due to the lack of high doses of steroids used in solid tumour regimens. However, many years after the treatment of nephro- or neuroblastoma, an excessive amount of body fat and characteristics of metabolic syndrome were associated with abdominal radiation [17]. Although people who receive abdominal radiation are also characterised by a more advanced stage of the disease and also receive more chemotherapy cycles than those with clinical tumour stage I or II. And still there is a significant difference between the therapy for nephroblastoma and, e.g., neuroblastoma stage IV treated with megachemotherapy and autologous HSCT.

Murphy and colleagues [18] performed a study similar to ours, but on a smaller group of patients. In their analysis, patients both during and after treatment had a higher percentage of fat compared to those in the control group. Interestingly, there were no statistically significant differences in body composition depending on the type of cancer in the group currently being treated. However, based on current data, it is not entirely clear which methods are best for cardiovascular risk assessment among cancer survivors. Specifically, the underestimation of obesity with BMI versus DXA may affect men exposed to abdominal/pelvic radiation. Furthermore, among former Children’s Oncology Group patients, overweight/obesity was found in 39% of subjects, but was not observed more frequently than in the control group without cancer [19]. Moreover, it is important to note the high prevalence of sarcopenic obesity during and after treatment for leukaemia limits the utility of BMI as an indicator of obesity [20].

Among the analysed factors affecting the development of overweight/obesity, we found radiation therapy and the use of allogeneic HSCT were important. Similarly, a previous study showed among 276 patients after completion of tumour treatment, 47.8% were overweight/obese after cranial radiation compared to 30.4% of those treated without radiation [21]. We did not notice differences in BMI, although patients treated with radiation had a higher percentage of body fat evaluated by electrical bioimpedance. Other authors showed the BMI and waist circumference were closely correlated with the dose of cranial radiation [22]. Perhaps the threshold that leads to the development of obesity after radiotherapy in ALL is a dose of ≥20 Gy [23]. Still, the question remains as to whether the omission of radiotherapy in modern ALL treatment in children reduces or completely eliminates the occurrence of excess body mass and body fat.

In our study, treatment with allogeneic HSCT was a risk factor for excessive body fat but not overweight/obesity. Similarly, Bizzarri *et al* [24] found a high proportion of patients with central obesity but normal BMI after HSCT due to hematologic malignancies, especially those who received total body irradiation and (in contrast to us) with a long time after transplantation. This phenomenon was associated with the lipodystrophic and sarcopenic phenotype of these patients [25]. Meanwhile, a long-term prospective follow-up of patients after allogeneic HSCT indicated that BMI decreases over time after transplantation, but this effect is more likely to be due to lean mass rather than fat mass loss [26]. Initial BMI prior to transplantation is a strong predictor of this index after transplantation, like in standard therapy. In another study after transplantation, high rates of central obesity and body fat were associated with the use of total body irradiation, but the presence of graft-versus-host disease resulted in less body fat [27].

In summary, we still do not know what contributes more to the occurrence of obesity after recovery from cancer – the disease itself or the treatment? The origin of obesity in cancer survivors is not fully explained, but likely includes physical inactivity, damage to the hypothalamus and endocrine organs, and resistance to insulin and leptin, among others (reviewed in [2]). In addition, the effect of clinical factors (including CNS irradiation and the use of glucocorticoids) on the occurrence of obesity after antineoplastic treatment has been shown to be stronger than that of genetic factors [28]. Some authors suggest obesity in patients after cancer treatment adversely affects their quality of life, including chemotherapy-induced peripheral neuropathy [29], while others found no relationship between quality of life after completed antitumour therapy and body weight [30].

The strength of our analysis is the creation of a regression model that explains which factors affect body mass, fat and muscle after treatment of childhood cancer. This model shows the factors that determine the occurrence of overweight/obesity and abnormal body composition in cancer survivors. The limitations of our study are the significant difference in time from the end of anticancer treatment between leukaemia/lymphoma and solid tumours groups, the small number of children included who were currently undergoing treatment of solid tumours, the difficulties with stratification according to glucocorticoid therapy, and a lack of prospective studies.

The occurrence of excessive body weight and body fat after cancer can and must be prevented. An early intervention based on family participation in patient lifestyle intervention has been proposed previously [31]. We recommend paediatricians and family physicians should actively detect and prevent cardiovascular disease in childhood cancer survivors.

## Conflicts of interest

The authors declare that they have no conflict of interest.

## Authors’ contributions

Małgorzata Sawicka-Żukowska and Włodzimierz Łuczyński equally contributed to this work. Małgorzata Sawicka-Żukowska and Maryna Krawczuk-Ryba designed the study, Małgorzata Sawicka-Żukowska and Włodzimierz Łuczyński were major contributors in writing the manuscript, Małgorzata Sawicka-Żukowska and Jakub Dobroch collected and analysed the patient’s data. All authors read and approved the final manuscript.

## Ethics approval and consent to participate

The study design was approved by the Ethics Committee at the Medical University of Bialystok in accordance with the Declaration of Helsinki (No 153-798883L). Signed informed consent was obtained from patients and their parents/guardians.

## Funding statement

The study was funded by the Medical University of Bialystok, Poland.

## Figures and Tables

**Table 1.** Number of patients, sex and age in examined and control subgroups.

	1 Leukaemias/lymphomas during therapy	2 Leukaemias/lymphomas after treatment termination	3 Solid tumours during therapy	4 Solid tumours after treatment termination	5 Control group	Statistical analysis
Number of patients	84	183	12	73	89	
Age of the assessment mean ± SD	8.22 ± 4.7	13.46 ± 4.0	9.81 ± 4.8	13.91 ± 4.9	13.38 ± 4.60	group 1 versus 2, 4, 5*** group 3 versus 2, 4, 5***
Sex: female/male N/N %/%	32/52 38.0%/62.0%	77/106 42.0%/58.0%	*7/5* 58.3%/41.7%	33/40 45.2%/54.8%	52/37 58.4%/41.5%	no statistical differences
Diagnosis	ALL, AML, HD, NHL	ALL, AML, HD, NHL	ES, OS, RMS	WT, NBL, RMS, ES, OS, GCTs, LCH	Healthy siblings, volunteers, hematologic disturbances suspected and excluded	
Age of diagnosis mean ± SD	7.44 ± 4.9	7.32 ± 4.5	9.03 ± 4.6	5.27 ± 4.7	-	group 3 versus 1,2,4* group 4 versus 1–3**
Time from treatment termination (median, 25–75 IQR)		4.38 ± 3.4		7.40 ± 4.7		***
Steroids in treatment N %	84 100%	166 90.7%	1 8.3%	6 8.2%	-	
Radiation in treatment N %	9 10.7%	62 33.9%	6 50.0%	37 50.7%	-	
Megachemotherapy in treatment N%	0 0.0%	51 27.9%	0 0.0%	0 0.0%	-	

ALL, acute lymphoblastic leukaemia; AML,acute myeloid leukaemia; HD, Hodgkin disease; NHL, non-Hodgkin lymphoma; RMS, rhabdomyosarcoma; LCH, Langerhans cell histiocytosis; NBL, neuroblastoma; OS, osteosarcoma; GCTs, germ cell tumours; WT, Wilms tumour; ES, Ewing sarcoma

* *p* = 0.01,

** *p* = 0.001,

*** *p* = 0.0001

**Table 2.** Standardised BMI and percentages of normal and abnormal weight in patients subgroups.

	1 Leukaemias/lymphomas during therapy	2 Leukaemias/lymphomas after treatment termination	3 Solid tumours during therapy	4 Solid tumours after treatment termination	5 Control group	Statistical analysis
BMI-SDS median (25–75 percentile)	0.38 (−0.61–1.39)	0.70 (−0.21–2.12)	−0.06 (−0.6–2.45)	0.07 (−0.55–0.59)	0.18 (−0.49–0.96)	group 2 versus 4** group 2 versus 5**
Underweight N %	10 11.9%	12 6.6%	2 16.7%	8 11.0%	5 5.6%	
Normal weight *N* %	45 53.6%	91 49.7%,	7 58.3%,	55 75.3%,	62 69.7%	group 1 versus 3–5** group 2 versus 3–5 **
Overweight N %	17 20.2%,	28 15.3%	0 0%	4 5.5%,	11 12.4%	
Obesity N %	12 14.3%	52 28.4%;	3 25%	6 8.2%;	11 12.4%	

** *p* = 0.001

**Table 3.** Percentages of patients with normal and abnormal amounts of fat or muscle mass in bioimpedance analysis.

	1 Leukaemias/lymphomas during therapy	2 Leukaemias/lymphomas after treatment termination	3 Solid tumours during therapy	4 Solid tumours after treatment termination	5 Control group	Statistical analysis
FAT g <N/N/>N	8.3%/45.2%/46.4%	16.9%/35.5%/47.5%	8.3%/66.6%/25%	24.6%/60.0%/15%	24.7%/51.6%/23.5%	group 2 versus 5 *
PBF % <N/N/>N	3.5%/30.9%/65.4%	9.2%/28.9%/61.7%	8.3%/33.3%/58.3%	13.6%/56.2%/30.1%	12.3%/57.3%/30.3%	group 2 versus 4* group 2 versus 5*
SMM <N/N/>N	61.9%/33.3%/4.8%	43.2%/49.2%/7.6%	91.6%/8.3%/0.0%	46.6%/50.7%/2.7%	35.9%/59.5%/4.5%	group 1 versus 5* group 3 versus 5*

N = normal

* *p* = 0.01

## References

[1] Bhatia S, Armenian SH, Armstrong GT (2015). Collaborative research in childhood cancer survivorship: the current landscape. J Clin Oncol.

[2] Teixeira JF, Maia-Lemos PD, Cypriano MD (2016). The influence of antineoplastic treatment on the weight of survivors of childhood cancer. J Pediatr (Rio J).

[3] Mostoufi-Moab S, Seidel K, Leisenring WM (2016). endocrine abnormalities in aging survivors of childhood cancer: a report from the Childhood Cancer Survivor Study. J Clin Oncol.

[4] Chemaitilly W, Cohen L (2017). Diagnosis of endocrine disease: endocrine late effects of childhood cancer and its treatments. Eur J Endocrinol.

[5] Felicetti F, Fortunati N, Brignardello E (2018). Cancer survivors: an expanding population with an increased cardiometabolic risk. Diabetes Res Clin Pract.

[6] Kulaga Z, Litwin M, Tkaczyk M (2010). The height-, weight-, and BMI-for-age of polish school-aged children and adolescents relative to international and local growth references. BMC Public Health.

[7] Tompuri TT, Lakka TA, Hakulinen M (2015). Assessment of body composition by dual-energy X-ray absorptiometry, bioimpedance analysis and anthropometrics in children: the Physical Activity and Nutrition in Children study. Clin Physiol Funct Imaging.

[8] Niewadzi E, Głowińska-Olszewska B, Łuczyński W (2013). Analysis of body composition with the use of bioimpedance in children with type 1 diabetes. Pediatr Endocrinol Diabetes Metab.

[9] Blundell JE, Dulloo AG, Salvador J (2014). Beyond BMI-phenotyping the obesities. Obes Facts.

[10] van Santen HM, Geskus RB, Raemaekers S (2015). Changes in body mass index in long-term childhood cancer survivors. Cancer.

[11] Jahnukainen K, Heikkinen R, Henriksson M (2015). Increased body adiposity and serum leptin concentrations in very long-term adult male survivors of childhood acute lymphoblastic leukemia. Horm Res Paediatr.

[12] Blijdorp K, van den Heuvel-Eibrink MM, Pieters R (2012). Obesity is underestimated using body mass index and waist-hip ratio in long-term survivors of childhood cancer. PLos ONE.

[13] Miller TL, Lipsitz SR, Lopez-Mitnik G (2010). Characteristics and determinants of adiposity in pediatric cancer survivors. Cancer Epidemiol Biomarkers Prev.

[14] Ness KK, DeLany JP, Kaste SC (2015). Energy balance and fitness in adult survivors of childhood acute lymhoblastic leukemia. Blood.

[15] Zhang FF, Rodday AM, Kelly MJ (2014). Predictors of being overweight or obese in survivors of pediatric acute lymphoblastic leukemia (ALL). Pediatr Blood Cancer.

[16] Asner S, Ammann RA, Ozsahin H (2008). Obesity in long-term survivors of childhood acute lymphoblastic leukemia. Pediatr Blood Cancer.

[17] van Waas M, Neggers SJ, Raat H (2012). Abdominal radiotherapy: a major determinant of metabolic syndrome in nephroblastoma and neuroblastoma survivors. PLos ONE.

[18] Murphy AJ, White M, Elliott SA (2015). Body composition of children with cancer during treatment and in survivorship. Am J Clin Nutr.

[19] Lindemulder SJ, Stork LC, Bostrom B (2015). Survivors of standard risk acute lymphoblastic leukemia do not have increased risk for overweight and obesity compared to non-cancer peers: a report from the Children’s Oncology Group. Pediatr Blood Cancer.

[20] Orgel E, Mueske NM, Sposto R (2016). Limitations of body mass index to assess body composition due to sarcopenic obesity during leukemia therapy. Leuk Lymphoma.

[21] Gunn HM, Emilsson H, Gabriel M (2016). Metabolic health in childhood cancer survivors: a longitudinal study in a long-term follow-up clinic. J Adolesc Youth Adult Oncol.

[22] Sohn YB, Kim SJ, Park SW (2011). The metabolic syndrome and body composition in childhood cancer survivors. Korean J Pediatr.

[23] Oeffinger KC, Mertens AC, Sklar CA (2003). Obesity in adult survivors of childhood acute lymphoblastic leukemia: a report from the Childhood Cancer Survivor Study. J Clin Oncol.

[24] Bizzarri C, Pinto RM, Ciccone S (2015). Early and progressive insulin resistance in young, non-obese cancer survivors treated with hematopoietic stem cell transplantation. Pediatr Blood Cancer.

[25] Wei C, Thyagiarajan MS, Hunt LP (2015). Reduced insulin sensitivity in childhood survivors of haematopoietic stem cell transplantation is associated with lipodystrophic and sarcopenic phenotypes. Pediatr Blood Cancer.

[26] Inaba H, Yang J, Kaste SC (2012). Longitudinal changes in body mass and composition in survivors of childhood malignancies after allogeneic hematopoietic stem-cell transplantation. J Clin Oncol.

[27] Ruble K, Hayat M, Steward KJ (2012). Body composition after bone marrow transplantation in childhood. Oncol Nurs Forum.

[28] Wilson CL, Liu W, Yang JJ (2015). Genetic and clinical factors associated with obesity among adult survivors of chidldhood cancer: a report from the St. Jude Lifetime Cohort. Cancer.

[29] Cox-Martin E, Trahan LH, Cox MG (2017). Disease burden and pain in obese cancer patients with chemotherapy-induced peripheral neuropathy. Support Care Cancer.

[30] Nayiager T, Anderson L, Cranston A (2017). Health-related quality of life in ling-term survivors of acute lymphoblastic leukemia in childhood and adolescence. Qual Life Res.

[31] Zhang FF, Parsons SK (2015). Obesity in childhood cancer survivors: call for early weight management. Adv Nutr.

